# Effects of lactation room quality on working mothers’ feelings and thoughts related to breastfeeding and work: a randomized controlled trial and a field experiment

**DOI:** 10.1186/s13006-022-00499-0

**Published:** 2022-08-09

**Authors:** Sjoukje A. van Dellen, Barbara Wisse, Mark P. Mobach

**Affiliations:** 1 Department of Psychology, University of Groningen, Groningen, The Netherlands; 2 NoorderRuimte, Research Centre for Built Environment, Hanze University of Applied Sciences, Groningen, The Netherlands; 3 Faculty of Management and Organization, The Hague University of Applied Sciences, The Hague, The Netherlands

**Keywords:** Breastfeeding, Lactation room quality, Theory of supportive design, Nursing facilities, Facility management, Environmental sensitivity, Stress reduction, Relaxation

## Abstract

**Background:**

The challenging combination of breastfeeding and work is one of the main reasons for early breastfeeding cessation. Although the availability of a lactation room (defined as a private space designated for milk expression or breastfeeding) is important in enabling the combination of breastfeeding and work, little is known about the effects of lactation room quality on mothers’ feelings and thoughts related to breastfeeding and work. We hypothesized that a high-quality lactation room (designed using the Theory of Supportive Design) would cause mothers to experience less stress, have more positive thoughts about milk expression at work, perceive more organizational support, and report more subjective well-being, than a low-quality lactation room.

**Methods:**

In an online randomized controlled trial (Study 1), Dutch mothers (*N* = 267) were shown either a high-quality or a low-quality lactation room (using pictures and descriptions for the manipulation) and were then asked about their feelings and thoughts. In a subsequent field experiment (Study 2) we modified the lactations rooms in a large organization in Groningen, the Netherlands, to manipulate lactation room quality, and asked mothers (*N* = 61) who used either a high-quality or low-quality lactation room to fill out surveys to assess the dependent variables.

**Results:**

The online study showed that mothers exposed to the high-quality lactation room anticipated less stress, more positive cognitions about milk expression at work, more perceived organizational support, and more subjective well-being than mothers exposed to the low-quality lactation room (*p* <  0.05). Moreover, the effect of lactation room quality on perceived organizational support was especially pronounced for mothers who were higher in environmental sensitivity. The field experiment showed that use of the high-quality room led to less reported stress than use of the low-quality room (*p* <  0.05). We also found that mothers who were higher in environmental sensitivity perceived more control over milk expression at work and experienced more subjective well-being in the high-quality condition than in the low-quality condition (*p* <  0.05).

**Conclusion:**

The current studies show that not only the availability, but also the quality of lactation rooms is important in facilitating the combination of breastfeeding and work.

**Supplementary Information:**

The online version contains supplementary material available at 10.1186/s13006-022-00499-0.

## Background

Research findings indicate that the challenging combination of breastfeeding and work is one of the main reasons for early breastfeeding cessation [1, 2]. To prevent mothers from having to choose between breastfeeding and career development, it is important to find new ways to better support breastfeeding mothers at work. While many factors play a role in creating a breastfeeding-friendly environment in the workplace, paid breastfeeding breaks and the availability of a lactation room (defined as a private space designated for milk expression or breastfeeding) are important basic requirements for enabling mothers to continue breastfeeding their babies when they return to work. However, related maternity protection legislation differs per country; the provision of paid breastfeeding breaks is included in the legislation of 71% of the countries worldwide, but the provision of a lactation room is included in the legislation of only 31% of countries [3]. Furthermore, legislation rarely offers any guidance related to the *quality* of nursing facilities.

In the Netherlands, breastfeeding rates are relatively low: the percentages of exclusive breastfeeding (operationalized as still receiving breast milk without receiving infant formula) and any breastfeeding at 6 months of age are 19 and 28% respectively [4]. Mandatory paid maternity leave in the Netherlands is 16 weeks, with a minimal of 10 weeks postnatal leave (Article 3.1, paragraph 1–3 of the *Labour and Care Act*). A breastfeeding mother is entitled to paid breastfeeding breaks during her workday until her infant is 9 months of age. The Dutch law furthermore states that an employer should provide a suitable, lockable, and private space for a breastfeeding employee (Article 4.8, paragraph 1 of the *Working Hours Act*), but does not further specify what suitable means in this context. A recent cross-sectional study conducted in the Netherlands showed that lactation room quality was generally low, and that lactation room quality was positively related to mothers’ satisfaction with the room and perceived ease of and support for milk expression at work [5].

**Fig. 1:**
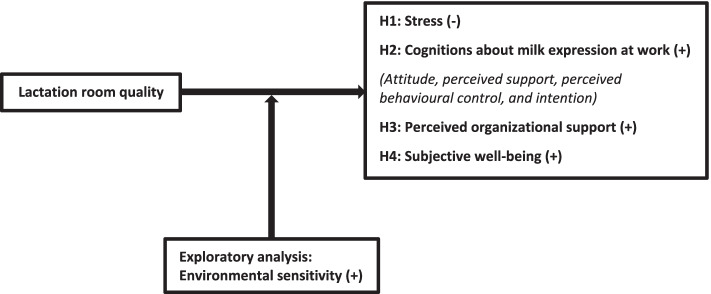
Conceptual model and hypotheses

Experimental research on the causal impact of lactation room quality on mothers’ thoughts and feelings related to milk expression at work has been lacking so far. Therefore, we conducted two experimental studies to investigate if the use of a high-quality (vs. low-quality) lactation room reduces mothers’ stress, and has a positive influence on their cognitions about milk expression at work, perceived organisational support, and subjective well-being. Lactation room quality was manipulated using the recommendations of the Theory of Supportive Design, which states that the built environment can have a psychological impact on individuals [6]. In addition, we explored the extent to which these effects are more pronounced in mothers who are higher in environmental sensitivity, since these mothers have the tendency to process stimuli and information strongly and deeply [7] (see Fig. 1). With this research we hope to uncover whether the provision of a high-quality lactation room can contribute to facilitating the combination of breastfeeding and work.

### The provision of lactation rooms to support breastfeeding

Breastfeeding women need to breastfeed or express milk regularly during the day in order to maintain milk supply and avoid medical problems related to a build-up of milk. For women who want to combine breastfeeding and work it is therefore important that measures are taken to enable breastfeeding or milk expression during working hours. Although there are several other options, such as allowing breastfeeding breaks at home or at the day-care, arguably the most common solution is to provide women with a lactation room at work where they can pump milk for their baby. Various studies have investigated if the presence of a lactation room can support breastfeeding by working mothers. A 2017 review found positive effects of access to a lactation space on breastfeeding initiation, breastfeeding duration, breastfeeding exclusivity, use of infant formula, predominant breastfeeding, and job satisfaction [8]. Yet, effects were not always strong and sometimes effects could only be found when certain conditions were met. For instance, one study found that while access to a lactation space did not have a significant effect on its own, the combination of an available lactation space *and* a refrigerator was associated with continued breastfeeding [9]. It may be that, apart from lactation room *availability*, lactation room *quality* is also important in predicting working mothers’ responses. Research on the effects of lactation room quality, however, is scarce. Moreover, guidelines on lactation room design are often limited to functional aspects. For instance, in the Netherlands the law states that a lactation room should be suitable, lockable, and private (Article 4.8, paragraph 1 of the *Working Hours Act*). Further directives explain that the lactation room should be lockable from the inside, it should have good hygiene and sufficient privacy, it should be sufficiently quiet and secluded, it should have a bed or couch, sufficient fresh air and climate control facilities, and there should be no risks involved (such as the presence of hazardous materials and contaminants) [10]. Although these basic functional requirements are a helpful starting point, they are not construed with the notion in mind that going above and beyond these basic aspects may have additional positive consequences for how mothers feel about combining breastfeeding and work. In the following, we will explain 1) how high-quality lactation rooms can be designed, and 2) why we think that lactation room quality may impact stress, cognitions about milk expression at work, perceived organizational support, and subjective well-being.

### Enhancing lactation room quality by applying the theory of supportive design

In line with the tenets of a recent cross-sectional study on lactation room quality [5], we posit that the quality of lactation rooms is determined by more than just basic functional aspects. Just like the quality of office rooms is enhanced by, for instance, indoor air quality, thermal comfort, lighting, acoustics, and natural, aesthetic and recreational aspects [11], the quality of lactation rooms is also dependent on more than the bare essentials required by legislation. A theory that provides guidance in how high quality lactations rooms can be designed is Ulrich’s Theory of Supportive Design [6]. The Theory of Supportive Design stems from a school of thought promoting evidence-based design in healthcare settings in order to create so-called ‘healing environments’. Literature reviews offer evidence that the built environment may indeed affect the health and well-being of users in healthcare settings [12–14]. Ulrich based his theory largely on the observation that, traditionally, the interior design of health facilities has emphasized only the functional delivery of healthcare, leading to facilities that may seem effective, but are also stressful because they don’t attend to the psychological needs of patients. The Theory of Supportive Design argues that more accommodating designs can reduce stress, by fostering perceptions of control, offering positive distraction, and encouraging social support [6]. Thus, to be considered high-quality, a room should address both psychological *and* functional needs. Applying these insights to lactation room design we argue that lactation rooms that incorporate the principles of the Theory of Supportive Design (by fostering perceptions of control, offering positive distraction, and encouraging social support) should be considered higher quality lactation rooms than rooms that do not incorporate these principles.

### Impact of lactation room quality on breastfeeding mothers

Based on the above, we first of all hypothesized that a high-quality lactation room, designed by the principles of the Theory of Supportive Design, will reduce mothers’ stress levels to a larger extent than rooms that are designed without adhering to those principles. Stress-reducing qualities may be particularly relevant for a lactation room, as stress has been shown to interfere with the release of oxytocin, a hormone responsible for the milk ejection reflex, and may thus lead to a disruption of the milk flow and a reduced milk volume, hence adversely affecting the process of breastfeeding [15–17]. Moreover, a recent review showed that stress reduction and relaxation can indeed help to improve breastfeeding outcomes [18]. Two recent studies that focused specifically on testing the tenets of Ulrich’s theory [6] showed that the greater the number of design features fostering perceptions of control, positive distraction, and social support, the lower patients’ perceived stress turned out to be [19, 20]. Although these studies focused on patients in hospital environments, we posit that these findings may apply to breastfeeding mothers in work settings as well. Therefore, we hypothesized that when mothers use a high-quality lactation room, they will experience lower stress levels than when they use a low-quality lactation room (Hypothesis 1, see Fig. 1).

Apart from reducing mothers’ stress levels, we hypothesized that high-quality lactation rooms may have additional beneficial effects, in particular on mothers’ thoughts related to milk expression at work. Evidence in this direction comes from a recent cross-sectional study that found an association between lactation room quality on the one hand and perceived behavioural control and perceived support for milk expression at work on the other [5]. In the current experimental study, we therefore examined the effects of lactation room quality on perceived behavioural control and perceived support for milk expression at work, and added two additional cognitions that are theoretically considered important in predicting behaviour [21]: attitude towards expressing milk at work, and intention to express milk at work. We hypothesized that when mothers use a high-quality lactation room, they will have more positive cognitions about milk expression at work than when they use a low-quality lactation room (Hypothesis 2, see Fig. 1).

Finally, we expect that lactation room quality may have an impact beyond mothers’ cognitions about milk expression at work. Since a lactating working mother spends several hours of her working week in a lactation room, lactation room quality may also affect cognitions that are not directly tied to breastfeeding and milk expression. In the current study, we focused in particular on whether lactation room quality influences mothers’ perceptions of organizational support and their subjective well-being. Perceived organizational support refers to the extent to which employees believe that the organization values their contribution and cares about their well-being, and has been shown to be positively related to favourable outcomes for employees (e.g., job satisfaction, positive mood) as well as organizations (e.g., affective commitment, performance, and lessened withdrawal behaviour [22]). A recent meta-analysis has shown that perceptions of family-supportive work practices are related to perceived organizational support, especially for those employees who need such practices [23]. Viewing the provision of high-quality breastfeeding facilities as family-supportive work practices, we hypothesized that when mothers use a high-quality lactation room, they will perceive more organizational support than when they use a low-quality lactation room (Hypothesis 3, see Fig. 1).

Subjective well-being refers to people’s cognitive and affective evaluations of their lives, or in other words, to the extent to which people are happy and satisfied with their lives [24]. Subjective well-being is associated with a wide spectrum of favourable outcomes, such as good health and longevity, better social relationships, creativity, and work performance [24]. A recent review has shown that a positive work-life balance is related to life satisfaction [25], which is one of the core components of subjective well-being [26]. Viewing high-quality breastfeeding facilities as a way of improving the work-life balance of breastfeeding employees, we hypothesized that when mothers use a high-quality lactation room, they will report more subjective well-being than when they use a low-quality lactation room (Hypothesis 4, see Fig. 1).

### Exploring the influence of individual differences in environmental sensitivity

When investigating the effect of environmental features on people, it is important to take into account that not all individuals may react equally to variations in the external environment. One variable that may be of particular importance in this regard is environmental sensitivity. Environmental sensitivity, measured as sensory processing sensitivity, is viewed as a fundamental trait and is defined as the degree to which an individual registers, processes, and responds to external stimuli [7, 27]. Whereas one person may be very sensitive to environmental influences, another may remain unperturbable under all circumstances. Although studies on the role of sensory processing sensitivity in environmental interventions are largely lacking, previous research with a precursory measure of environmental sensitivity – i.e., stimulus screening and arousability [28] – showed that this variable could moderate the effects of environmental interventions. For example, it was found that stimulus screening and arousability moderated people’s stress, arousal, and cognitive appraisals of a room in reaction to colour-use in a simulated hospital room [29] as well as workers’ productivity in reaction to colour-use in office settings [30], indicating that people high in stimulus screening and arousability show stronger reactions to environmental interventions. Based on this research, we expect that the effects of lactation room quality will be stronger to the extent that mothers are higher in environmental sensitivity (see Fig. 1). Given the lack of direct empirical support for this notion, we will investigate whether this is the case in exploratory moderation analyses.

In sum, we aimed to investigate the influence of lactation room quality on working mothers’ feelings and thoughts related to breastfeeding and work. We hypothesized that a high-quality lactation room will reduce mothers’ stress, and have a positive influence on their cognitions about milk expression at work, perceived organisational support, and subjective well-being. In addition, we expected these effects to be more pronounced to the extent that mothers are higher in environmental sensitivity. We used a mixed-methods research design and tested our hypotheses in two methodologically complementary studies. We used an online randomized controlled trial to minimize threats to internal validity (Study 1) and a field experiment to improve the ecological validity of the research findings (Study 2).

## Methods

### Study 1: a randomized controlled trial

#### Design and participants

Study 1 was set up as a randomized controlled trial, which is considered the golden standard for testing causal claims, because it minimizes threats to internal validity [31]. A total of 267 Dutch mothers participated in an online study that employed a 1 × 2 (lactation room quality: high versus low) between-subjects experimental design. Mothers were randomly assigned to either the high-quality lactation room condition (*n* = 136) or the low-quality lactation room condition (*n* = 121), using pictures and descriptions for the manipulation of lactation room quality. Environmental sensitivity was added to the design as a continuous variable. Inclusion criteria were: (1) current or previous experience with breastfeeding and (2) being employed. Exclusion criteria were: (1) not meeting the inclusion criteria, and (2) age and/or completion time deviating more than 3 SD from the mean. The mothers had a mean age of 32.5 years (SD = 4.3), and worked on average 27.1 hours per week (SD = 6.8).

#### Procedure

Mothers were recruited through a message on the Facebook page of a popular Dutch website with breastfeeding information and were informed that breastfeeding and/or parenting books would be raffled among the participants who completed the survey. All mothers provided their informed consent before initiating the survey. First, we assessed environmental sensitivity, then mothers were randomly assigned to either the high-quality or the low-quality lactation room condition. They were shown pictures and a description of either the high-quality or the low-quality lactation room, and asked to imagine a scenario where they made use of this lactation room to express milk. After viewing the pictures and reading the description, they answered the survey questions comprising a manipulation check, the dependent variables, and demographic items.

#### Manipulation of lactation room quality

**Fig. 2:**
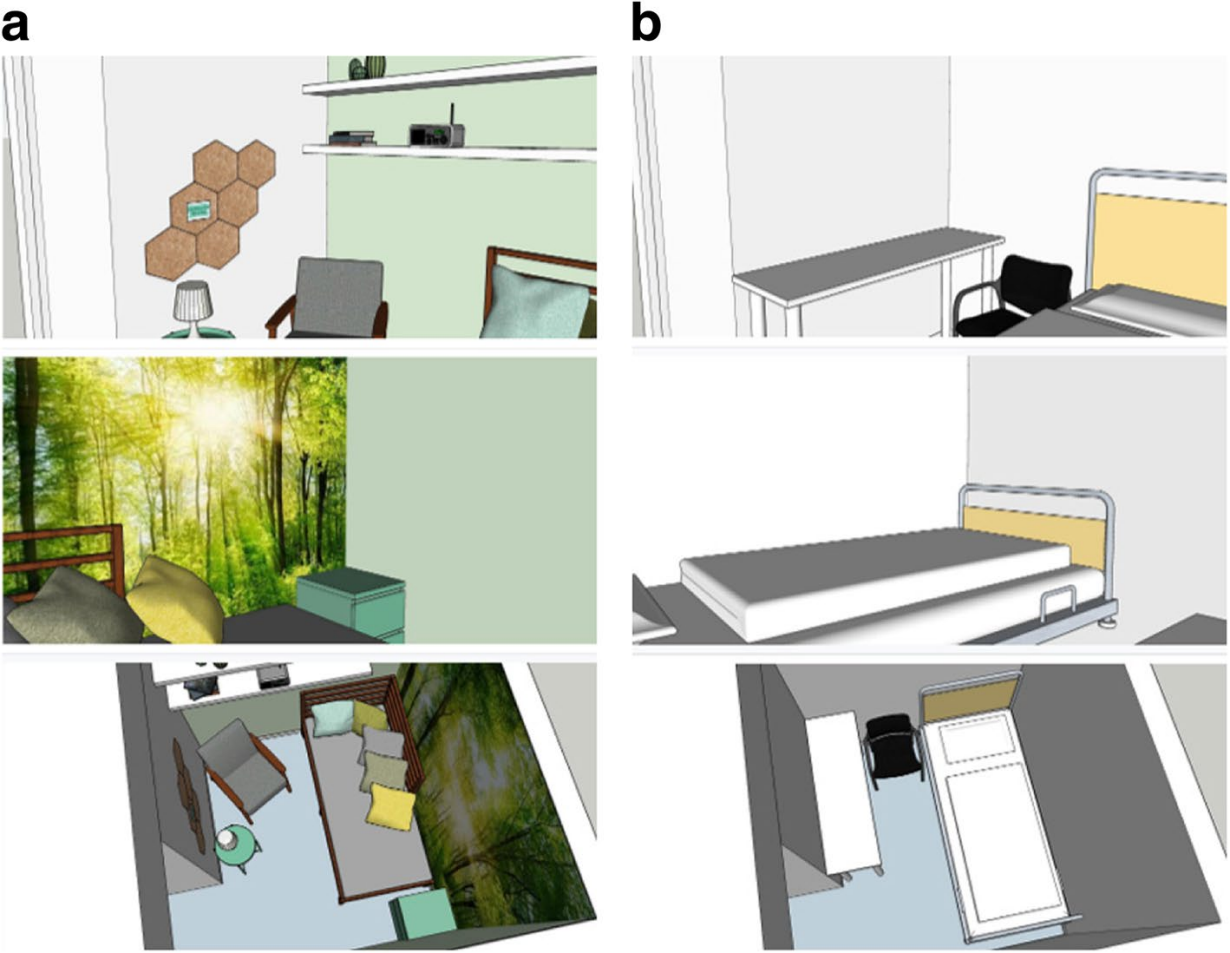
**a** Drawings of the high-quality lactation rooms. **b** Drawings of the low-quality lactation rooms

The manipulation of lactation room quality was based on the premise that a high-quality lactation room should not only meet the basic functional requirements, but should also follow the recommendations from the Theory of Supportive Design [6]. The stimulus materials for the high-quality and the low-quality lactation room conditions consisted of design drawings created by a professional interior designer, accompanied by a matching description of the room. The design drawings of the low-quality lactation room were based on examples of existing Dutch lactation rooms that only met the minimum requirements for lactation rooms according to Dutch law and guidelines, but did not foster perceptions of control, positive distraction, or social support. These design drawings showed a white room, containing a chair, a table, and a hospital bed. The design drawings of the high-quality lactation room met the minimum requirements, and in addition they aimed at fostering perceptions of control (e.g., adjustable lighting and pillows), positive distraction (e.g., nature images and decoration), and social support (e.g., supportive messages about breastfeeding). The drawings in this condition showed a room decorated with green paint on one wall and a forest-photo-wallpaper on another wall, containing a comfortable chair, a table, a bed, and many decorations, such as: pillows, a mood light, a bulletin board, books, ceramic plants, a radio etc. Both design drawings were accompanied by the following text: *‘Below you can see images of one lactation room from three different viewpoints, and a list of the available facilities. Study these images and the accompanying text carefully. Imagine expressing milk in such a room; try to imagine what this would feel like.’* For the low-quality lactation room, the text proceeded as follows: *‘This lactation room contains the following: A chair, a table for the breast pump, and a bed. There is also an adjoining room with a sink, and a door with a lock.’* In contrast, for the high-quality lactation room the text proceeded as follows: *‘This lactation room contains the following: a chair, a table for the breast pump, a bed with pillows, a mood light, a bulletin board, a card with the text: ‘Good that you are here! Take your time’, two shelves, a breastfeeding book, two picture books with nature images, a radio and 3 ceramic plants, wallpaper with an image of sun rays in the forest, a cabinet with two drawers. There is also an adjoining room with a sink, and a door with a lock.’* Because the study took place during the COVID-19 pandemic, we added information in both conditions about hygienic measures (indicating that the room is cleaned daily and that water and soap, paper towels, hygienic wipes, and disinfecting hand gel are also provided). See Fig. 2a and b for the design drawings.

##### Manipulation check

To verify that our manipulation of lactation room quality based on the Theory of Supportive Design was successful, we developed a 4-item scale. Items were: ‘This room contains images of nature’, ‘This room contains nice, beautiful, or interesting things’, ‘This room is adjustable to my needs’, ‘This room makes me feel supported in milk expression at work’. Mothers were asked to indicate their agreement on a seven-point Likert scale from (1) ‘totally disagree’ to (7) ‘totally agree’ (α = .79). Furthermore, we asked mothers to award a report grade for lactation room quality on a scale of 1 to 10 (1 = very bad; 10 = very good). As intended, one-way ANOVAs showed that mothers perceived the high-quality lactation room as being more consistent with the Theory of Supportive Design (*M*_*=*_ 6.01, *SD* = 0.68) than the low-quality lactation room condition (*M* = 3.13, *SD* = 0.88, *F*(1,265) = 893.60, *p* <  0.001). Moreover, mothers awarded a higher report grade for lactation room quality in the high-quality lactation room condition (*M*_*=*_ 8.93, *SD* = 1.14) than in the low-quality lactation room condition (*M* = 6.69, *SD* = 1.57, *F*(1,265) = 981,01, *p* <  0.001). We therefore conclude that our manipulation of lactation room quality was successful.

#### Measures

##### Environmental sensitivity

Environmental sensitivity was assessed before participants saw the design drawings and consisted of the 12-item short version of the HSP Scale [7, 32]. Example items of the HSP-scale are ‘Do you notice and enjoy delicate or fine scents, tastes, sounds, works of art?’ and ‘Are you bothered by intense stimuli, like loud noises or chaotic scenes?’ Answering options ranged from 1 ‘not at all’ to 7 ‘extremely’. The internal consistency of the scale was good (α = .82).

##### Stress

Anticipated stress was measured using the short version of the State-Trait Anxiety Inventory for adults [33]; this short version [34] is well validated and has been shown to correlate highly with physiological measures of stress [35]. Mothers could indicate on a four-point Likert scale, ranging from (1) ‘not at all’, to (4) ‘very much so’ the extent to which they would feel calm/ tense/ upset/ relaxed/ content/ worried in the room that was shown to them (α = .82).

##### Cognitions about milk expression at work

Anticipated attitude, perceived support, and perceived behavioural control towards milk expression at work were operationalized according to the guidelines by Ajzen [21, 36]. Attitude was measured by presenting mothers with the following statement: ‘For me expressing milk at work in the room that was shown would be…’. This statement was followed by three 7-point, semantic, differential adjective scales: ‘unenjoyable – enjoyable, unpleasant – pleasant, negative – positive’ (α = .94). Perceived support was measured with four bipolar items: ‘Judging from the room that was shown I think that my supervisor approves of me expressing breast milk at work’ and ‘Judging from the room that was shown I think that my supervisor supports me expressing breast milk at work’. These two items were then repeated, replacing ‘my supervisor’ with ‘my co-workers’. All of the items were answered using a 7-point Likert scale, ranging from (1) ‘strongly disagree’ to (7) ‘strongly agree’ (α = .93). Perceived behavioural control was measured by two items: ‘In the room that was shown, expressing milk at work would be…for me’, rated on a scale from (1) ‘impossible’ to (7) ‘possible’, and ‘In the room that was shown, I could express milk at work if I wanted to’, rated on a scale from 1 ‘strongly disagree’ to 7 ‘strongly agree’ (α = .61). Anticipated intention to express milk at work was measured with a single item, based on an Australian study on breastfeeding duration [37]. The item was: ‘How long would you like to express milk at work if the lactation room shown was available at work? In that case, I would like to express milk at work until my baby is ... months old’. Participants were asked to indicate their intended duration of milk expression at work as a whole number of months.

##### Perceived organizational support

Perceived organizational support was measured by selecting eight high-loading items (loadings from .71 to .84) from the Survey of perceived organizational support [38]. Examples of items that were used are: ‘The organization fails to appreciate any extra effort from me’ (reversed), ‘The organization really cares about my well-being’, ‘The organization cares about my general satisfaction at work’, ‘The organization shows very little concern for me’ (reversed). The statements were preceded by the sentence: ‘Taking into account the room that was shown I would think that…’. Participants indicated their agreement with each item using a 7-point Likert-type scale (1) ‘strongly disagree’, (7) ‘strongly agree’ (α = .92).

##### Subjective well-being

Subjective well-being was measured based on the 2-item scale developed by Statistics Netherlands [39]. The items were: ‘On a scale from 1 to 10 can you indicate to what extent you would consider yourself to be a happy person if you expressed milk in the room that was shown? (1 = completely unhappy, 10 = completely happy)’ and ‘On a scale from 1 to 10 can you indicate how satisfied would you be with the life you lead at the moment if you expressed milk in the room that was shown? (1 = completely dissatisfied and 10 = completely satisfied)’ (α = .93).

### Study 2: a field experiment

#### Design and participants

To complement the results of Study 1 and improve the ecological validity of our research findings, a second experimental study was conducted in a real-life setting. A total of 61 lactating employees from a large hospital in Groningen, the Netherlands, participated in the research. Since on average 90 mothers make use of the lactation rooms on the maternity ward each year according to the secretary of the ward (Mollema, Y., personal communication, August 15, 2017), 61 participants over a two-year period reflects a response rate of approximately 34%. We used a 1 × 2 (lactation room quality: high versus low) between-subjects experimental design, with two measurement points: the first (T1) as soon as the mother returned to work (or maximally four weeks afterwards), and the second (T2) four weeks after their return to work (thereby making sure mothers could have used the lactation room for at least four weeks). Although the intention was that mothers filled in the T1 questionnaire as soon as they returned to work, most mothers signed up somewhat later. It was decided that the T1 questionnaire could be filled in maximally four weeks after the return to work. Environmental sensitivity was added to the design as a continuous variable. Inclusion criteria were: (1) returning from maternity leave no more than 4 weeks prior to T1, and (2) making use of the lactation rooms on the maternity ward of the hospital at work at T1. Exclusion criteria were: (1) no longer making use of the respective lactation rooms at work at T2. The experiment took place over a two-year period: from June 2018 until June 2020. In the first year, all participating mothers were assigned to the low-quality lactation room condition (*n* = 32) and in the second year all participating mothers were assigned to the high-quality lactation room condition (*n* = 29). The mothers had a mean age of 31.5 years (*SD* = 3.1) and worked on average 30.3 hours per week (*SD* = 7.1). On average mothers used the lactation room 5.8 times per week (*SD* = 2.8). About two thirds of the mothers (62.7%) also used an alternative lactation room (*M* = 3.4 times per week; *SD* = 2.4). There were no significant differences between mothers in the experimental group and the control group with regard to these characteristics.

#### Procedure

Mothers were recruited by placing flyers in the three lactation rooms in the maternity ward at the hospital. The flyers pointed out that participants for a study on experiences with milk expression at work were sought and that breastfeeding and/or parenting books would be raffled among the participants who completed the survey. Mothers could receive further information and an invitation to participate, by leaving their name, e-mail, and the date they had returned from maternity leave on a participation form. Every mother who handed in the participation form (at the front desk of the maternity ward), received a chocolate bar as a token of our gratitude. Invitations for the pre-test questionnaire were sent as soon as the mothers signed up for the study, mostly in the first week after they returned to work. Invitations for the post-test questionnaire were sent four weeks after the mothers returned to work. We emphasized that participation in the study was anonymous and voluntary and that they could withdraw from the study at any time. All mothers provided their informed consent before continuing to the survey. In the pre-test, mothers answered survey questions about their environmental sensitivity and demographic information. In the post-test, when mothers had been using the hospital’s lactation room for at least four weeks, they answered survey questions comprising a manipulation check and the dependent variables.

#### Manipulation of lactation room quality

**Fig. 3:**
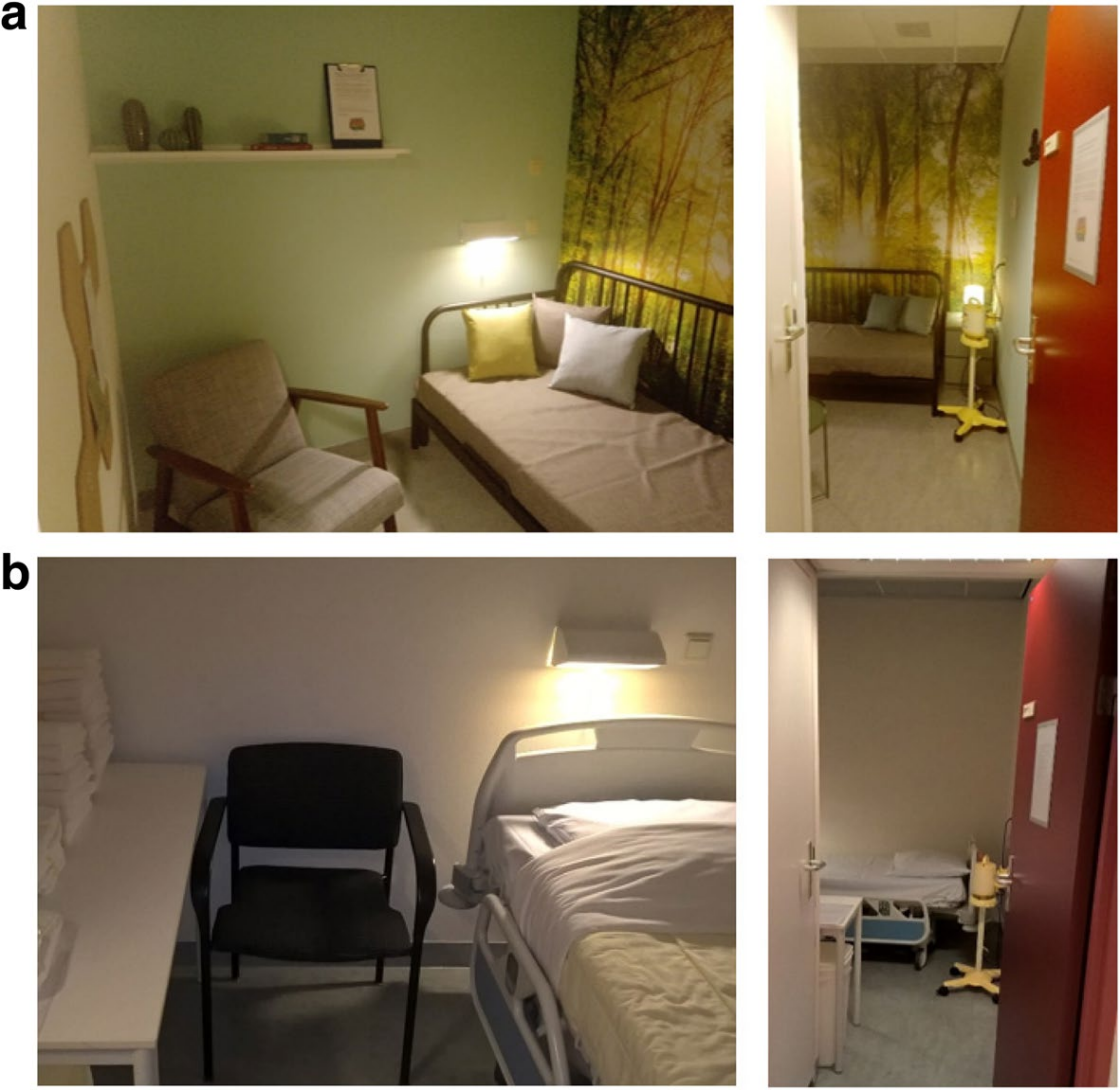
**a** Photos of the high-quality lactation rooms. **b** Photos of the low-quality lactation rooms

The manipulation of lactation room quality corresponded to that in Study 1, but in the field experiment, we used and adapted the existing lactation rooms in the maternity ward of the hospital. In the low-quality condition, mothers made use of three identical standard lactation rooms in the hospital maternity wards where the research took place. These low-quality lactation rooms were basic white hospital rooms, containing a chair, a table, a hospital bed, and a hospital grade breast pump (which prevented unwanted individual variance in pumping experiences due to the breast pump used.) After one year the three lactation rooms were refurbished and painted in order to create the high-quality condition, based on the design drawings that had been created for Study 1. Similar to Study 1, these high-quality lactation rooms were identically decorated with green paint on one wall and a forest-photo-wallpaper on another wall, they contained a comfortable chair, a table, a bed with multiple pillows, a mood light, a bulletin board, with a card that welcomed mothers to the lactation room, a breastfeeding information book, two picture books with nature images, ceramic plants, a cabinet with two drawers, and a hospital grade breast pump. For photographs of the lactation rooms in the high-quality and the low-quality condition, see Fig. 3a and b.

##### Manipulation check

The manipulation checks (α = .86 for the 4-item scale) were measured exactly as in Study 1. As intended, one-way ANOVAs showed that mothers perceived the high-quality lactation room as being more consistent with the Theory of Supportive Design (*M* = 5.45, *SD* = 0.90) than the low-quality lactation room condition (*M* = 2.28, *SD* = 0.86, F(1,59) = 196.18, *p* <  0.001). Moreover, mothers awarded a higher report grade for lactation room quality in the high-quality lactation room condition (*M*_*=*_ 7.79, *SD* = 0.94) than in the low-quality lactation room condition (*M* = 6.22, *SD* = 1.52, F(1,59) = 23.12, p <  0.001). We therefore conclude that our manipulation of lactation room quality was again successful.

#### Measures

The measures we used corresponded to the ones we used in Study 1. We made some small adjustments in wording, taking into account that this was a field study instead of a scenario study. This, for instance, allowed us to use the present tense (e.g., I feel) instead of the conditional simple tense (e.g., I would feel).

Environmental sensitivity (α = .81) was measured exactly as in Study 1. Stress (α = .81), subjective well-being (α = .76), and perceived organizational support (α = .90) were measured using the same items as in Study 1, but stated in the present tense. To assess attitude (α = .88), perceived support (α = .87), and perceived behavioural control (α = .77) towards milk expression at work we used similar measures as in Study 1. However, we specified the behaviour of ‘expressing milk at work’ further by adding ‘until my baby is at least 6 months old’. Moreover, for the measurement of attitude we added two semantic, differential adjective scales: ‘worthless – valuable’ and ‘useless – useful’, to also include utilitarian aspects of attitude [40]. For perceived behavioural control we added 2 items to improve the reliability of the scale: ‘For me pumping milk at work until my baby is at least 6 months old is…’, rated on a scale from 1 ‘hard’ to 7 ‘easy’, and ‘It is mostly up to me whether or not I pump milk at work until my baby is at least 6 months old’, rated on a scale from 1 ‘strongly disagree’ to 7 ‘strongly agree’. We replaced the intention to express milk at work of Study 1 with a 3-item measure based on the guidelines developed by Ajzen [21]. Answer options were on a scale from (1) ‘strongly disagree’ to (7) ‘strongly agree’. The items were: ‘I intend to express milk at work until my baby is at least 6 months old’, ‘I will do my best to express milk at work until my baby is at least 6 months old’, and ‘I plan to express milk at work until my baby is at least 6 months old’ (α = .94).

## Results

### Study 1

One-way ANOVAs were performed to test the hypotheses. A *p*-value of 0.05 was considered significant (*p* <  0.020 after applying Holm-Bonferroni correction to reduce the chance of a type I error). First, as hypothesized, mothers anticipated to experience less stress when the lactation room was high-quality rather than low-quality (see Table 1). Furthermore, mothers that were presented a lactation room that was high-quality as compared to low-quality anticipated to have a more positive attitude towards expressing milk at work, to perceive more support from managers and coworkers, and to have more behavioural control towards expressing milk at work. Finally, mothers in the high-quality lactation room condition anticipated to perceive a higher level of organizational support, and to experience a higher level of subjective well-being than did mothers in the low-quality lactation room condition. Contrary to expectations, the intended duration of breastfeeding did not differ for mothers presented with the high-quality or low-quality lactation room condition.

**Table 1:** One-way ANOVA reports the effect of lactation room quality on the dependent variables (*N* = 267)

	High-quality lactation room		Low-quality lactation room			
Dependent Variable	*M*	SD	*M*	SD	F	*p*
Stress	1.31	0.33	1.83	0.44	122.72	< 0.001*
Attitude	6.23	1.07	4.71	1.36	103.10	< 0.001*
Perceived support	5.93	1.12	5.06	1.41	31.05	< 0.001*
Perceived behavioural control	6.63	0.85	6.31	1.12	6.76	< 0.01*
Intention	14.84	5.54	14.88	5.91	0.00	n.s.
Perceived organizational support	6.29	0.74	5.03	1.23	103.16	< 0.001*
Subjective well-being	8.55	1.01	6.93	1.57	101.14	< 0.001*

*Note*: df = 1, df error = 265 for all seven tests. *Significant at *p* < 0.020

### Exploratory analyses of the moderating role of environmental sensitivity

**Fig. 4:**
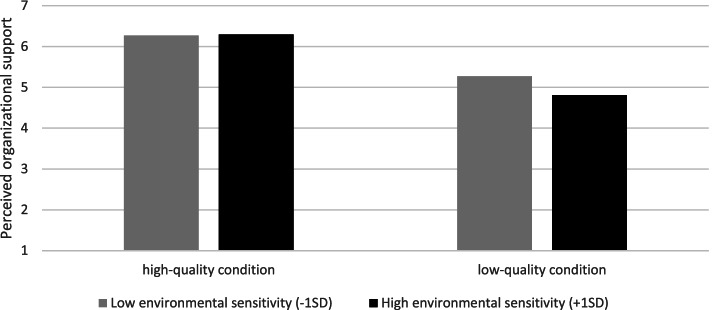
Moderating effect of environmental sensitivity in the relationship between lactation room quality and perceived organizational support

Hayes Process macro [41] (model 1) was used to test whether environmental sensitivity moderated the effect of lactation room quality on each of our dependent measures. A *p*-value of 0.05 was considered significant (we decided not to apply a Holm-Bonferroni correction in these exploratory analyses, because we did not want to increase the chance of a type II error because of the exploratory nature of the analyses). For the main effects, we found that in the high-quality condition mothers anticipated less stress (*b* = − 3.13, *t* = − 11.02, *p* <  0.001), a more positive attitude towards milk expression at work (*b* = 1.49, *t* = 10.13, *p* <  0.001), more support from managers and coworkers (*b* = .86, *t* = 5.50, *p* <  0.001), more behavioural control towards expressing milk at work (*b* = .31, *t* = 2.58, *p* <  0.01), more organizational support (*b* = 1.25, *t* = 10.12, *p* <  0.001), and more subjective well-being (*b* = 1.60, *t* = 9.99, *p* <  0.001) than in the low-quality condition. Furthermore, we found that as mothers scored higher on environmental sensitivity, they anticipated more stress (*b* = .58, *t* = 2.43, *p* < 0.05), a less positive attitude towards milk expression at work (*b* = −.36, *t* = − 2.92, *p* < 0.01), less organizational support (*b* = −.25, *t* = − 2.47, *p* < 0.05), and less subjective well-being (*b* = −.30, *t* = − 2.24, *p* < 0.05). Apart from these main effects, we also found a significant interaction effect of lactation room quality and environmental sensitivity on perceived organizational support (*b* = .27, *t* (263) = 1.98, *p* < 0.05). Simple slopes analysis [42] showed that there was a significant positive relationship between lactation room quality and perceived organizational support when environmental sensitivity was both low (− 1 *SD*; *b =* 1.00, *t* = 5.69, *p* < 0.001) and high (+ 1 *SD*, *b =* 1.49, *t* = 8.56, *p* < 0.001), but that the effect was stronger in the latter case. This means that, in line with our expectations, the effect of lactation room quality on perceived organizational support was especially pronounced for mothers who are high in environmental sensitivity, see Fig. 4. No other significant interaction effects were found.

### Study 2

One-way ANOVAs were performed to test the hypotheses; again, a *p*-value of 0.05 was considered significant (*p* < 0.021 after applying Holm-Bonferroni correction to reduce the chance of a type I error). As hypothesized, mothers experienced less stress when the lactation room was high-quality rather than low-quality (see Table 2). Although other main effects were in the expected direction, they were not significant. During the last few months of this research the COVID-19 pandemic reached the Netherlands. To rule out that these circumstances influenced the results, we also analyzed the data while excluding those mothers that filled out questionnaires during the COVID-19 pandemic. This reduced the sample to 55 participants; the conclusions flowing from the analysis remained the same as with the larger sample of 61 participants.

**Table 2:** One-way ANOVA reports the effect of lactation room quality on the dependent variables (*N* = 61)

	High-quality lactation room		Low-quality lactation room			
Dependent Variable	*M*	SD	*M*	SD	F	*p*
Stress	1.50	0.39	1.86	0.52	9.40	< 0.01*
Attitude	5.75	0.84	5.62	1.00	0.31	n.s.
Perceived support	5.47	1.38	5.14	1.24	0.94	n.s.
Perceived behavioural control	5.53	1.19	5.48	0.96	0.03	n.s.
Intention	6.63	0.51	6.10	1.63	2.78	n.s.
Perceived organizational support	4.62	1.16	4.27	0.99	1.55	n.s.
Subjective well-being	8.36	0.69	8.11	0.82	1.67	n.s.

*Note*: df = 1, df error = 59 for all seven tests. *Significant at *p* < 0.021

### Exploratory analyses of the moderating role of environmental sensitivity

Hayes Process macro [41] (model 1) was used to test whether environmental sensitivity moderated the effect of lactation room quality on each of our dependent measures. A *p*-value of 0.05 was considered significant (again, we decided not to apply a Holm-Bonferroni correction here, because we did not want to increase the chance of a type II error). For the main effects, we found that in the high-quality condition mothers anticipated less stress (*b* = − 2.15, *t* = − 3.03, *p* < 0.01) than in the low-quality condition. Furthermore, we found that as mothers scored higher on environmental sensitivity, they experienced less subjective well-being (*b =* −.46, *t* = − 2.47, *p* < 0.05). Apart from these main effect, we also found two interaction effects.

**Fig. 5:**
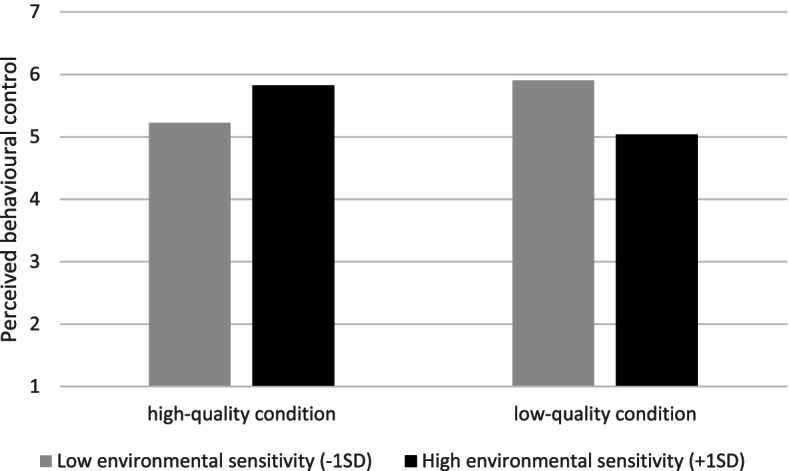
Moderating effect of environmental sensitivity in the relationship between lactation room quality and perceived behavioural control

First, we found a significant interaction effect of lactation room quality and environmental sensitivity on perceived behavioural control (*b =* .82, *t* (57) = 2.57, *p* < 0.05). Simple slopes analysis showed that there was a significant positive relationship between lactation room quality and perceived behavioural control when environmental sensitivity was high (+ 1 *SD*, *b =* .79, *t* = 2.01, *p* < 0.05), which was not the case when environmental sensitivity was low (− 1 *SD*, *b =* −.68, *t* = − 1.75, *p =* .09). This means that the positive effect of lactation room quality on perceived control was only present for mothers who are high in environmental sensitivity, see Fig. 5.

**Fig. 6:**
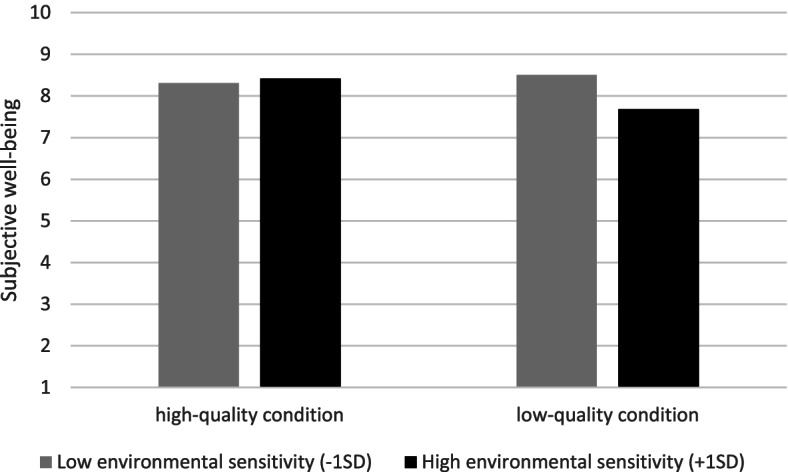
Moderating effect of environmental sensitivity on the relationship between lactation room quality and subjective well-being

Second, we found a significant interaction effect of lactation room quality and environmental sensitivity on subjective well-being (*b =* 0.52, *t* (57) = 2.30, *p* < 0.05). Simple slopes analysis showed that there was a significant positive relationship between lactation room quality and subjective well-being when environmental sensitivity was high (+ 1 *SD, b =* 0.73, *t* = 2.63, *p* < 0.05), which was not the case when environmental sensitivity was low (− 1 *SD, b =* − 0.20, *t* = −.73, *p =* .47). This means that the positive effect of lactation room quality on subjective well-being was only present for mothers who are high in environmental sensitivity, see Fig. 6. No other significant interaction effects were found.

## Discussion

In the current paper, we reported two methodologically complementary experiments, both examining the effects of lactation room quality on mothers’ feelings and thoughts. In Study 1, an online scenario study, we found that mothers exposed to a high-quality lactation room anticipated less stress, more positive cognitions about milk expression at work, more perceived organizational support, and more subjective well-being than mothers exposed to a low-quality lactation room. Also, we found that environmental sensitivity moderated the effect of lactation room quality on perceived organizational support. Specifically, we found that the positive effect of lactation room quality on perceived organizational support was more pronounced for mothers higher in environmental sensitivity. In Study 2, a field experiment, we replicated some, but not all of the findings. Importantly, we again found that mothers who used the high-quality room experienced less stress than mothers who used the low-quality room. Moreover, although we did not find significant main effects for other dependent variables in Study 2, we did find significant interaction effects of lactation room quality and environmental sensitivity on perceived behavioural control and subjective well-being. That is, mothers who scored higher on environmental sensitivity, experienced more behavioural control and subjective well-being in the high-quality condition than in the low-quality condition. Mothers who scored lower on environmental sensitivity were not affected by the quality of the lactation room with respect to experienced control and subjective well-being.

Our aim with this research was to uncover whether the provision of a high-quality lactation room could help to facilitate the combination of breastfeeding and work, and our results suggest that this is indeed the case, especially so for mothers higher in environmental sensitivity. The fact that Study 2 had less significant results as compared to Study 1, could be explained by the fact that people sometimes overestimate the extent to which certain prospective events or conditions will impact their responses (a so-called impact bias [43]). Because Study 1 assessed mothers’ anticipated responses to hypothetical lactation rooms (that is, they saw *drawings* of rooms and were asked to *imagine* making use of that room), they may have underestimated the extent to which other factors may also influence their stress-levels and cognitions (so-called focalism [43]), and they may have overestimated the effect of lactation room quality. Given the generally weaker effects in Study 2, mothers may indeed have overestimated the effects of lactation room quality to some extent in Study 1. However, another potentially relevant factor may be that the sample size in Study 2 was limited to 61 participants. As such, the statistical power was on the low side, and this may have hampered the obtainment of significant results.

Our findings have several theoretical implications. Importantly, we found support for the expected positive effect of lactation room quality on mothers’ stress-levels (Hypothesis 1) in both studies. This confirms the tenets of the Theory of Supportive Design, stating that a design that fosters perceptions of control, offers positive distraction, and encourages social support can reduce stress levels [6] - as corroborated in two previous studies [19, 20]. Even though the Theory of Supportive Design was originally developed as a framework to study how design can be supportive to patients in a hospital setting [19, 20], the current findings show that the theory can be usefully applied to the design of lactation rooms as well. Possibly, the Theory of Supportive Design can be applied to an even broader range of settings than originally envisioned, most notably to settings in which promoting relaxation is desirable (such as lactation rooms, dental practice waiting rooms, or wellness and meditation rooms within organizations). To our knowledge, there is only one previous study that also focused on design as a means of mitigating stress in breastfeeding mothers [44], although not in a work-setting. This study examined the experiences of breastfeeding mothers with a so-called Snoezelen room in a hospital [44]. The room included moving images, music, and aromatherapy, and was evaluated very positively by the participating mothers. Furthermore, most mothers were able to achieve breastfeeding in the room, despite previous breastfeeding problems [44]. Although this was a qualitative study, and consisted of only a small sample (*N* = 11), it confirms our current experimental findings, indicating that a high-quality environment can positively affect mothers’ stress levels and facilitate breastfeeding.

Another key finding of our research is that lactation room quality (by itself or in conjunction with environmental sensitivity) affects mothers’ cognitions related to milk expression at work (Hypothesis 2). We found mothers’ positive attitude towards and perceived support of milk expression at work was higher in the high-quality than in the low-quality lactation room (Study 1). Moreover, mothers’ perceived behavioural control with respect to milk expression at work was also positively affected by lactation room quality (Study 1), particularly for mothers high in environmental sensitivity (Study 2). The current study therefore corroborates and extends the findings of a previous cross-sectional study showing that lactation room quality was positively related to mothers’ cognitions about milk expression at work [5]. Interestingly, we did not find any effects of lactation room quality on intention to express milk at work in either study; however, this might be due to a ceiling effect, as the intended duration in Study 1 was already high (on average 15 months), compared to the relatively low breastfeeding rates in the Netherlands [4].

In addition, in Study 1 we found that lactation room quality affects mothers’ perceived organizational support and subjective well-being (Hypothesis 3 and 4), and that latter finding was also found in Study 2 for mothers high in environmental sensitivity. Therefore, it seems that providing a high-quality lactation room can have positive consequences even for factors that are not directly related to breastfeeding. Although previous studies have indicated that perceptions of family-supportive work practices and a positive work-life balance are positively related to perceived organizational support and subjective well-being [23, 25], this is the first study to link lactation room quality to these important outcomes. The fact that we only found effects on perceived organizational support in Study 1, and not Study 2, can have several reasons. Apart from the earlier mentioned potential effects of impact bias in Study 1, the influence of other forms of organizational support (e.g., direct emotional support by colleagues and managers) could have been relatively strong in Study 2, thus reducing the relative effects of lactation room quality. Another possibility is that the participants attributed any supportive influence of the high-quality lactation room to the researchers instead of to their organization, due to the fact that the participants were aware that they were taking part in research. Nonetheless, these findings are highly relevant, as perceived organizational support and subjective well-being are linked to a myriad of positive outcomes for organizations and employees, such as job satisfaction, positive mood, affective commitment, performance, and lessened withdrawal behaviour (perceived organizational support [22]), and good health and longevity, better social relationships, creativity, and work performance (subjective well-being [24]).

A final theoretically important finding is that environmental sensitivity moderated the effect of lactation room quality on several dependent measures in both Study 1 and 2. These findings are in line with previous research, showing that people high in environmental sensitivity respond more strongly to interventions [29, 30, 45]. Our findings testify to the importance of taking this variable into account in research on (environmental) interventions, because it allows for a better understanding of the effectiveness and efficiency of such interventions within certain sub-groups of people. Moreover, since employees who are high in environmental sensitivity are particularly sensitive to stressors [45], they are an important potential target group for organizational interventions focused on preventing mental health problems and improving well-being among employees.

### Strengths, limitations and directions for future research

A major strength of the current research is that we used methodological triangulation to test our hypotheses. Although all methods have their own strengths and weaknesses, limitations of individual methods can be mitigated by using triangulation in so-called mixed methods research. This is considered to be valuable as it helps to show the robustness of findings across different research methods [46]. In our research, we used an online randomized controlled trial to minimize threats to internal validity (Study 1) and a field experiment to improve the ecological validity of the research findings (Study 2). By using methodological triangulation to investigate the effects of lactation room quality on mothers’ feelings and thoughts, we were able to show that various findings were not limited to one study (taking away concern that findings may potentially partially be explained by bias resulting from used methods) and therefore we provide stronger evidence and support for the conclusions of our research.

Another strength of the current research is that our lactation room design manipulations, based on the Theory of Supportive Design [6], were studied in a field experiment. Previous studies using the Theory of Supportive Design were either laboratory studies [19] or field studies that were observatory rather than experimental in nature [20]. Therefore, the fact that rooms designed according to the insights from the Theory of Supportive Design yielded positive effects in a real-life setting, testifies to the applicability of the theory. However, a potential limitation of our field experiment is that we were not able to control all factors. Specifically, our design was such that we first researched the effects of the low-quality room (in year 1), and then, after remodelling, researched the effects of the high-quality room (in year 2). Although seasonal effects were controlled for in this set-up (we gathered participants for each condition during one whole year), our results may have been impacted by changes or events that occurred during the two years we ran this study. One important event in this regard was the COVID-19 pandemic that started during the end of year two of our study. However, we found that when we excluded mothers who participated during the COVID-19 period our conclusions flowing from the analysis remained the same, which strengthens our confidence in our findings. Nonetheless, other potential changes or events may in principle play a role. Future research may therefore replicate our study using a design in which participants for both conditions are gathered in the same time frame. Another useful suggestion would be to incorporate virtual reality techniques into the research designs. Virtual reality allows for more controlled circumstances than a field experiment, while at the same time increasing possibilities to recruit a larger sample. Moreover, given that virtual reality offers the enhanced capacity for an immersive, interactive experience with the design [47], it may be easier for participants to imagine oneself in a certain situation than with the use of scenario studies.

An important limitation of the current study is that while we examined the effects of lactation room quality on the feelings and thoughts of breastfeeding mothers, we did not examine the downstream effects on behavioural outcomes, such as the duration of breast milk expression and breastfeeding. Notably, previous research underscores the importance of mothers’ stress and cognitions for breastfeeding practices. For example, a recent review showed that stress reduction and relaxation interventions can indeed help to improve breastfeeding outcomes [18], and several studies suggest that maternal cognitions are important predictors of milk expression and breastfeeding behaviour [48–50]. Future research could fruitfully examine the effects of lactation room quality on (long-term) behavioural outcome measures, such as breast milk expression and breastfeeding duration, and investigate if these effects are mediated by feelings and thoughts of breastfeeding mothers. Moreover, future studies could also consider adding physiological outcome measures, such as breast milk volume and composition (e.g., fat content), and, for example, physiological measures of stress (e.g., cortisol level, heart rate, blood pressure, and fingertip temperature). Furthermore, it would be interesting to study the effects of lactation room quality, in combination with other methods of relaxation-enhancement, such as meditation [18]. Finally, although the current study focused on the impact of lactation room quality as an independent factor, creating a breastfeeding-friendly work environment goes beyond the provision of a high-quality lactation room. Future studies could therefore examine the impact of a composite program of family-friendly measures, including paid parental- and sick leave, breastfeeding support, affordable child care, flexible work arrangements, and high-quality breastfeeding facilities. This would help to paint a broader picture of the critical role that organizations play in enabling women to continue breastfeeding upon their return to work.

### Practical implications

For organizations it is important to realize that offering good breastfeeding facilities creates a win-win situation, benefitting not only mothers and babies, but organizations as well. Since breastfeeding improves the health and well-being of infants and mothers [51], it can lead to reduced sick leave and health care costs. Moreover, breastfeeding support at work can lead to higher job satisfaction, a better work-life balance [52], and may even reduce staff turnover [53]. As such, facilitating breastfeeding in the workplace is a highly relevant topic to facility management practices, not only to respect diversity and stimulate inclusiveness, but also to foster a healthier workplace. The current research offers important insight into what organizations can do to facilitate mothers in combining breastfeeding and work. To support organizations in implementing high-quality lactation rooms, it would be useful to further explore practical organizational issues of costs and benefits, occupancy rates, and possibilities for multi-functional use of spaces, as well as to help raise awareness of the multiple value creation resulting from the provision of high-quality breastfeeding facilities.

The current study highlights the importance of the quality of the breastfeeding facilities that organizations offer for lactating mothers’ feelings and thoughts. Moreover, the current study provides clear guidelines that organizations can use in lactation room design: a high-quality lactation room should not only include the basic functional requirements as currently outlined in legislation and government guidelines [10], but should also address psychological needs, by fostering perceptions of control, offering positive distraction, and encouraging social support, as outlined in the Theory of Supportive Design [6].

## Conclusion

The ability of mothers to combine work and breastfeeding successfully offers important societal benefits due to the important long term health benefits for mothers as well as children [51]. While many factors play a role in creating a breastfeeding-friendly environment in the workplace, the availability of a lactation room is an important prerequisite for enabling mothers to continue breastfeeding when they return to work. The current study shows that not only the availability, but also the quality of lactation rooms is important in facilitating the combination of breastfeeding and work. The inclusion of quality guidelines for breastfeeding facilities in organisations’ family-friendly policies could therefore further expand and secure much-needed support for breastfeeding workers.

## Supplementary Information

**Additional file 1.** Data study 1.

**Additional file 2.** Syntax study 1.

**Additional file 3.** Data study 2.

**Additional file 4.** Syntax study 2.

## Data Availability

All of the data generated or analysed during this study are included in this published article and in the supplementary information files.

## References

[1] Rollins NC, Bhandari N, Hajeebhoy N, Horton S, Lutter CK, Martines JC (2016). Why invest, and what it will take to improve breastfeeding practices?. Lancet..

[2] Odom EC, Li R, Scanlon KS, Perrine CG, Grummer-Strawn L (2013). Reasons for earlier than desired cessation of breastfeeding. Pediatrics..

[3] Addati L, Cassirer N, Gilchrist K (2014). Maternity and paternity at work: law and practice across the world.

[4] van Dommelen P, Engelse O. Peiling melkvoeding van zuigelingen in 2018 [poll milk nutrition of infants in 2018]. Tijdschrift voor de Jeugdgezondheidszorg [J Youth Health Care]. 2021:53. 10.1007/s12452-021-00251-w.

[5] van Dellen SA, Wisse B, Mobach MP, Albers CJ, Dijkstra A (2021). A cross-sectional study of lactation room quality and Dutch working mothers' satisfaction, perceived ease of, and perceived support for breast milk expression at work. Int Breastfeed J.

[6] Ulrich RS (1991). Effects of interior design on wellness: theory and recent scientific research. J Health Care Inter Des.

[7] Aron EN, Aron A (1997). Sensory-processing sensitivity and its relation to introversion and emotionality. J Pers Soc Psychol.

[8] Dinour LM, Szaro JM (2017). Employer-based programs to support breastfeeding among working mothers: a systematic review. Breastfeed Med.

[9] Amin RM, Said ZM, Sutan R, Shah SA, Darus A, Shamsuddin K (2011). Work related determinants of breastfeeding discontinuation among employed mothers in Malaysia. Int Breastfeed J.

[10] Ministerie van Sociale Zaken en Werkgelegenheid [Ministry of Social Affairs and Employment] (2018). Kolven op het werk: dit zijn de richtlijnen [pumping at work: these are the guidelines].

[11] Al Horr Y, Arif M, Kaushik A, Mazroei A, Katafygiotou M, Elsarrag E (2016). Occupant productivity and office indoor environment quality: a review of the literature. Build Environ.

[12] Huisman ER, Morales E, van Hoof J, Kort HS (2012). Healing environment: a review of the impact of physical environmental factors on users. Build Environ.

[13] Salonen H, Lahtinen M, Lappalainen S, Nevala N, Knibbs LD, Morawska L (2013). Physical characteristics of the indoor environment that affect health and wellbeing in healthcare facilities: a review. Intell Build Int.

[14] Ulrich RS, Zimring C, Zhu X, DuBose J, Seo HB, Choi YS (2008). A review of the research literature on evidence-based healthcare design. HERD..

[15] Dewey KG (2001). Maternal and fetal stress are associated with impaired lactogenesis in humans. J Nutr.

[16] Lau C (2001). Effects of stress on lactation. Pediatr Clin N Am.

[17] Ueda T, Yokoyama Y, Irahara M, Aono T (1994). Influence of psychological stress on suckling-induced pulsatile oxytocin release. Obstet Gynecol.

[18] Gómez L, Verd S, de la Banda G, Cardo E, Servera M, Filgueira A (2021). Perinatal psychological interventions to promote breastfeeding: a narrative review. Int Breastfeed J.

[19] Andrade CC, Devlin AS (2015). Stress reduction in the hospital room: applying Ulrich's theory of supportive design. J Environ Psychol.

[20] Andrade CC, Devlin AS, Pereira CR, Lima ML (2017). Do the hospital rooms make a difference for patients’ stress? A multilevel analysis of the role of perceived control, positive distraction, and social support. J Environ Psychol.

[21] Ajzen I (1991). The theory of planned behavior. Organ Behav Hum Decis Process.

[22] Rhoades L, Eisenberger R (2002). Perceived organizational support: a review of the literature. J Appl Psychol.

[23] Kurtessis JN, Eisenberger R, Ford MT, Buffardi LC, Stewart KA, Adis CS (2017). Perceived organizational support: a meta-analytic evaluation of organizational support theory. J Manag.

[24] Diener E, Oishi S, Tay L (2018). Advances in subjective well-being research. Nat Hum Behav.

[25] Sirgy MJ, Lee DJ (2018). Work-life balance: an integrative review. Appl Res Qual Life.

[26] Linley PA, Maltby J, Wood AM, Osborne G, Hurling R (2009). Measuring happiness: the higher order factor structure of subjective and psychological well-being measures. Personal Individ Differ.

[27] Pluess M (2015). Individual differences in environmental sensitivity. Child Dev Perspect.

[28] Mehrabian A (1977). Individual differences in stimulus screening and arousability. J Pers.

[29] Dijkstra K, Pieterse ME, Pruyn AT (2008). Individual differences in reactions towards color in simulated healthcare environments: the role of stimulus screening ability. J Environ Psychol.

[30] Kwallek N, Soon K, Lewis CM (2007). Work week productivity, visual complexity, and individual environmental sensitivity in three offices of different color interiors. Color Res Appl.

[31] Andrade C (2018). Internal, external, and ecological validity in research design, conduct, and evaluation. Indian J Psychol Med.

[32] Pluess M (2013). Sensory processing sensitivity: a potential mechanism of differential susceptibility. Society for Child Development (SRCD) biennial meeting.

[33] Spielberger CD, Gorsuch RL, Lushene R, Vagg PR, Jacobs GA (1983). Manual for the state-trait anxiety inventory.

[34] Marteau TM, Bekker H (1992). The development of a six-item short-form of the state scale of the Spielberger state-trait anxiety inventory (STAI) [published correction appears in Br J Clin Psychol. 2020;59(2):276]. Br J Clin Psychol.

[35] Arora S, Tierney T, Sevdalis N, Aggarwal R, Nestel D, Woloshynowych M (2010). The Imperial stress assessment tool (ISAT): a feasible, reliable and valid approach to measuring stress in the operating room. World J Surg.

[36] Ajzen I (2002). Constructing a TPB questionnaire: conceptual and methodological considerations.

[37] Blyth RJ, Creedy DK, Dennis CL, Moyle W, Pratt J, De Vries SM (2004). Breastfeeding duration in an Australian population: the influence of modifiable antenatal factors. J Hum Lact.

[38] Eisenberger R, Huntington R, Hutchison S, Sowa D (1986). Perceived organizational support. J Appl Psychol.

[39] van Beuningen J, van der Houwen K, Moonen L (2014). Measuring well-being: an analysis of different response scales.

[40] Batra R, Ahtola OT (1991). Measuring the hedonic and utilitarian sources of consumer attitudes. Mark Lett.

[41] Hayes AF (2017). Introduction to mediation, moderation, and conditional process analysis: a regression-based approach.

[42] Aiken LS, West SG (1991). Multiple regression: testing and interpreting interactions.

[43] Wilson TD, Gilbert DT (2005). Affective forecasting: knowing what to want. Curr Dir Psychol Sci.

[44] Hauck YL, Summers L, White E, Jones C (2008). A qualitative study of Western Australian women's perceptions of using a Snoezelen room for breastfeeding during their postpartum hospital stay. Int Breastfeed J.

[45] Greven CU, Lionetti F, Booth C, Aron EN, Fox E, Schendan HE (2019). Sensory processing sensitivity in the context of environmental sensitivity: a critical review and development of research agenda. Neurosci Biobehav Rev.

[46] Turner SF, Cardinal LB, Burton RM (2017). Research design for mixed methods: a triangulation-based framework and roadmap. Organ Res Methods.

[47] Dunston PS, Arns LL, Mcglothlin JD, Lasker GC, Kushner AG, Wang X, Tsai JJH (2011). An immersive virtual reality mock-up for design review of hospital patient rooms. Collaborative design in virtual environments.

[48] Bai Y, Middlestadt SE, Peng CY, Fly AD (2010). Predictors of continuation of exclusive breastfeeding for the first six months of life. J Hum Lact.

[49] Bai YK, Dinour LM, Pope GA (2016). Determinants of the intention to pump breast milk on a university campus. J Midwifery Womens Health.

[50] Guo JL, Wang TF, Liao JY, Huang CM (2016). Efficacy of the theory of planned behavior in predicting breastfeeding: meta-analysis and structural equation modeling. Appl Nurs Res.

[51] Victora CG, Bahl R, Barros AJ, França GV, Horton S, Krasevec J (2016). Breastfeeding in the 21st century: epidemiology, mechanisms, and lifelong effect. Lancet..

[52] Jantzer AM, Anderson J, Kuehl RA (2018). Breastfeeding support in the workplace: the relationships among breastfeeding support, work-life balance, and job satisfaction. J Hum Lact.

[53] Ortiz J, McGilligan K, Kelly P (2004). Duration of breast milk expression among working mothers enrolled in an employer-sponsored lactation program. Pediatr Nurs.

